# 661. Quality vs. Quantity: Improving How EMR Chat Features Are Utilized Amongst the Burn Team

**DOI:** 10.1093/jbcr/irag033.498

**Published:** 2026-04-06

**Authors:** Cassandra O'Rourke

**Affiliations:** Rhode Island Burn Center, Providence, RI

## Abstract

**Introduction:**

The electronic medical record (EMR) has made the accessibility of patient information efficient and provides additional pathways for inter burn team communication through the use of chat features within the EMR. The chat feature within the EMR can help ensure information is attached to a specific patient to improve accuracy, however the ease of use can make this feature be overutilized. The excessive use of EMR chat creates notification fatigue, patient care issues can be missed or ignored, and needing to frequently respond to the chats can take time away from direct patient care. In an effort to decrease the unnecessary, excessive use of the EMR chat feature, a plan was developed by the burn team to address this concern.

**Methods:**

Initially, 3 months of data was collected to determine the number of EMR chats/patient/day that occurred amongst the burn team members. A 3 phase plan was developed to proactively decrease the number of EMR chats amongst the team members. Phase 1 was initiating the use of a "sticky note." This note contains simple bullet points regarding the patient's care plan, updated daily M - F with more information on Friday to anticipate weekend needs. This information included highlights such as dressing plan, surgical dates, therapy guidelines, and anticipated discharge plan/date. Two months later, Phase 2 was started. This phase was the creation of two EMR chat burn groups, adult and pediatric. Chats were to be sent to the group, not individual team members. The final phase was initiated 2 months later, providing professional, constructive feedback when the chat feature was unnecessarily or inappropriately used. This plan was developed and extensively discussed and presented weekly at burn huddle with all disciplines present prior to initiating Phase 1.

**Results:**

Three months prior to the initiation of Phase 1, the burn team EMR chats peaked at 31.6 EMR chats/patient/day. Two months after the initiation of Phase 1, the EMR chats decreased to 19.6 EMR chats/patient/day. The EMR chats decreased further 2 months after the initiate of Phase 2 to 17.9 EMR chats/patient/day. Now six months after the phases were initiated, the EMR chats are continuing to decrease to 16.2 EMR chats/patient/day.

**Conclusions:**

The EMR offers many advantages for inter team communication, however over use can have negative impacts on the burn team members efficiency and trickle down to interfere with direct patient care and patient safety. A simple, well communicated plan to effectively and appropriately utilize the EMR chat feature can significantly decrease the number of EMR chats/patient/day improving patient care and efficiency of burn team communication.

**Applicability of Research to Practice:**

Decreasing the number of EMR chats received by every team member increases time at the bedside and prevent errors due to distraction and signal fatigue. Further studies could be designed to look at hospital safety occurrences prior to and after the initiation of a communication improvement program.

**Funding for the study:**

N/A.

